# A Fast and Effective System for Detection of Neonatal Jaundice with a Dynamic Threshold White Balance Algorithm

**DOI:** 10.3390/healthcare9081052

**Published:** 2021-08-16

**Authors:** Wei-Yen Hsu, Han-Chang Cheng

**Affiliations:** 1 Department of Information Management, National Chung Cheng University, Chiayi 621, Taiwan; shet4937563@gmail.com; 2 Center for Innovative Research on Aging Society, National Chung Cheng University, Chiayi 621, Taiwan; 3 Advanced Institute of Manufacturing with High-Tech Innovations, National Chung Cheng University, Chiayi 621, Taiwan

**Keywords:** neonatal jaundice detection, automatic white balance, dynamic threshold, a detection algorithm

## Abstract

Neonatal jaundice is caused by high levels of bilirubin in the body, which most commonly appears within three days of birth among newborns. Neonatal jaundice detection systems can take pictures in different places and upload them to the system for judgment. However, the white balance problem of the images is often encountered in these detection systems. The color shift images induced by different light haloes will result in the system causing errors in judging the images. The true color of images is very important information when the detection system judges the jaundice value. At present, most systems adopt specific assumption methods and rely on color charts to adjust images. In this study, we propose a novel white balance method with dynamic threshold to screen appropriate feature factors at different color temperatures iteratively and make the adjustment results of different images close to the same. The experimental results indicate that the proposed method achieves superior results in comparison with several traditional approaches.

## 1. Introduction

Jaundice is caused by excessive bilirubin content in the human body [1] and causes yellow or green pigmentation in the skin or sclera. Bilirubin is a byproduct of the breakdown of old red blood cells and can cause skin irritation, white feces, and dark urine. Newborns with jaundice usually show symptoms within 3 days after delivery, but most patients recover naturally without complications. However, newborns with extremely high bilirubin content or jaundice for an extended period can develop kernicterus [2,3]. Neonatal jaundice is a common disease among newborns, approximately 84% of whom experience it [4]. Newborns with health conditions may exhibit bilirubin levels lower than the normal level. To prevent fatal brain damage, jaundice diagnosis entails precise physical examinations, which may require blood testing or specific medical equipment. Consequently, performing jaundice diagnosis away from the hospital is nearly impossible. In most newborns, the bilirubin content peaks within a few days after discharge from the hospital [5,6]; therefore, visual observation of neonatal jaundice symptoms by parents is the most common detection method. However, research has suggested that even the most experienced physicians cannot accurately identify neonatal jaundice using unaided vision [7]. Therefore, remote healthcare platforms may be used to acquire information and advice when performing neonatal jaundice diagnosis at home [2].

Bhutani prepared a nomogram to illustrate the differences in newborns’ bilirubin levels with time after birth [8]; the figure also indicates the normal range of newborn bilirubin content over time. Other studies have verified methods for assessing jaundice risk in newborns [9]; such methods include the measurements of total serum bilirubin (TSB) and transcutaneous bilirubin (TcB). TSB measurement is an invasive method that directly evaluates the bilirubin content in blood samples. TSB measurement is currently considered the most accurate method and is the standard for assessing jaundice risk. By contrast, TcB was designed specifically for noninvasive equipment and indirectly measures bilirubin content. Healthcare personnel can place a TcB measurement device on the forehead or chest of a newborn. The device emits a specific wavelength to determine the skin reflection and absorbance rates, which are then used to determine the bilirubin level [7].

This study explored the use of digital applications for neonatal jaundice detection. Many applications and platforms based on health detection have been put forth successively in recent years [10,11], and the application of intelligent devices to health detection is becoming increasingly universal [12]. Remote healthcare applications currently available focus mainly on auxiliary applications to assist users in everyday life [13,14] and support applications to monitor patients’ physical conditions and provide them with health advice [15,16]. However, remote healthcare applications have several limitations, including privacy concerns during data transmission and differences in the functions of professional healthcare equipment. In addition, such applications cannot fully satisfy patients’ autonomy requirements when resting at home. Addressing these shortcomings is crucial for developing remote healthcare schemes [17,18]. Regarding the use of digital applications in remote healthcare, various novel topics have received increasing attention, such as improving communication functions and components [19,20], long-term health care applications [21], integration of cloud databases [22], and the development of artificial intelligence technology [23].

In this study, an effective white balance method was developed for adjusting different color temperatures, which could be used in the white balance process of a jaundice detection system. The proposed method was used in the jaundice detection process to contribute to the accuracy of subsequent detection. Images were captured in different room lights with different color temperatures, in order to prove the adjustability of the method at different color temperatures. The quality and quantity of the images captured at different color temperatures were compared with those found using traditional methods.

## 2. Materials and Methods

**Figure 1:**
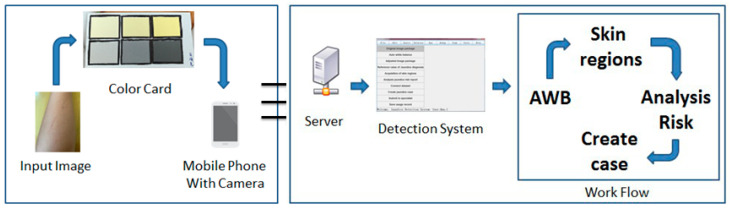
Detection system preprocessing flow chart.

For the adjustment of different color temperatures in neonatal jaundice detection, a white balance method was designed to adjust the images at different color temperatures to provide a nearly consistent color temperature. We performed a series of evaluations on the performance and quality of the adjusted images according to the neonatal jaundice image data sets collected by the doctors. The main contribution of the proposed method is that it can reduce the impact of different color temperatures on the images in the image preprocessing stage of the detection system, so as to minimize the subsequent error of the detection system in capturing the jaundice characteristics of the image. This method also allows researchers and ordinary neonatologists to reduce the impact of color shifts when initially judging and detecting neonatal jaundice from images. Figure 1 shows the pre-operation of the jaundice detection system and the preprocessing procedure of the back-end system.

### 2.1. Materials

The study recruited 38 jaundiced neonates. The 38 participants were photographed at six different color temperatures (28 K, 32 K, 40 K, 48 K, 56 K, 65 K), and 38 sets of images at different color temperatures were obtained for a total of 228 images. The skin of infants with high bilirubin levels is more yellowish than that with low bilirubin levels, and it also behaves differently when adjusted under different light sources. Therefore, we divided the sample composition into high risk and low risk groups according to reference [1]. Table 1 lists the distribution of bilirubin levels and the number of group samples for high risk and low risk groups, where the bilirubin level 15 (mg/dL) of newborns is used as the benchmark. This study only involved participants who were at either a low risk or a high risk of developing jaundice. The study was approved by the Institutional Review Board of Chiayi Christian Hospital’s Ditmanson Medical Foundation and complied with the Declaration of Helsinki [24]. The guardians of the participants provided advised written consent, and they were informed of the right to quit the study at any time. The participants were put in a crib, and the photographic equipment (an iPhone 6) was fixed for shooting at the same distance. The indoor ambient light was simulated by a lamp tube with varying color temperatures (28 K, 32 K, 40 K, 48 K, 56 K, 65 K). The white balance method for adjusting the images and the data analysis program were developed using Python (Anaconda Spyder).

### 2.2. Proposed White Balance Method

**Figure 2:**
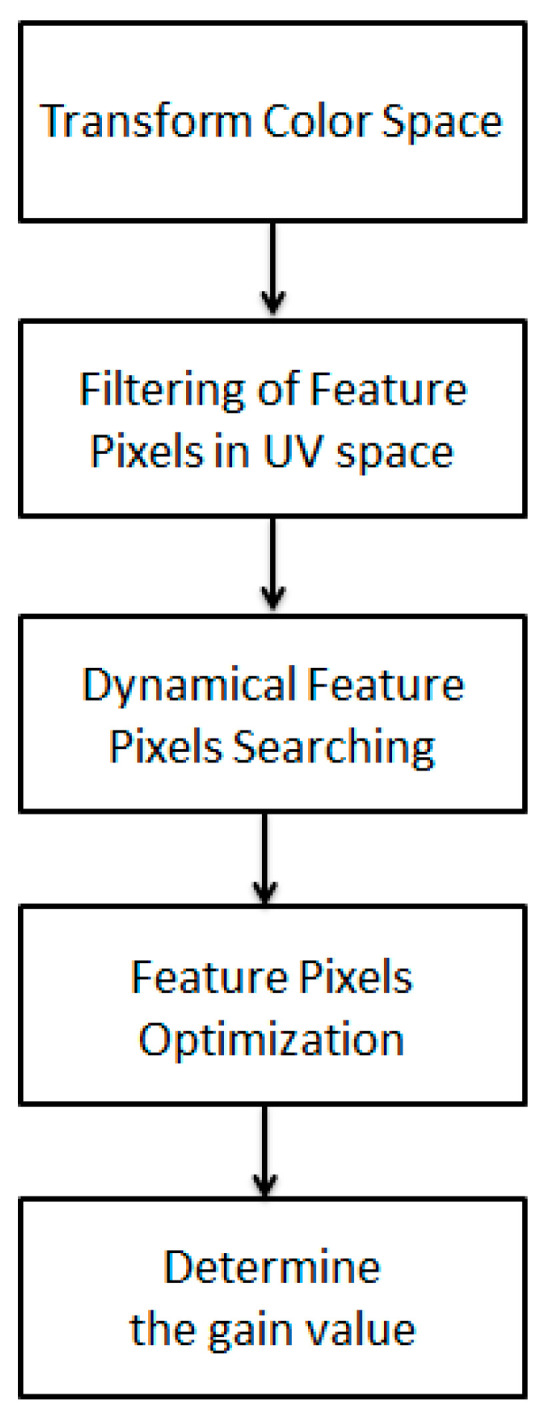
Flow chart of white balance method.

After a series of tests and data collection, the weights threshold and percentage threshold parameters were set up. The eligible image gain value was judged according to the image preprocessing and feature extraction. The screening range and feature selection were used as the threshold, in which the weights threshold was the threshold of the screening range in the color range of the image and was used to select the suitable factor for adjusting colors in the image color temperature environment (generally 0.5 to 1.5). The percentage threshold was the threshold for selecting the quantity from numerous candidate feature factors and was used to screen the reserved feature factors that matched the condition of the weights threshold, so as to reduce the probability of excessively dispersed adjustments resulting from excessive factors (generally 0.2 to 0.001). Finally, each eligible feature factor was used as the gain value after iterative correction, and the original image was adjusted. The flow chart of proposed white balance method is shown in Figure 2.

#### 2.2.1. Filtering of Feature Pixels in UV Space

According to the color deviation of the light source mentioned by dynamic threshold theory [25], it is basically generated in color space, and the brightness value of the light source color cannot be obtained in an RGB space. In the YUV space, Y represents the brightness value, while UV represents the chromatic value of red and blue, respectively. Color deviation problems usually refer to low color temperature (warm color system) and high color temperature (cool color system) problems; a low color temperature causes images to be inclined to red, while a high color temperature causes images to be inclined to blue. Therefore, the image was mapped onto the UV channel of the YUV space, so as to analyze the color of the image light source.

The average absolute differences ($D_{u}$ and $D_{v}$) of the image in the UV channel were calculated as the image center position, after which the pixels within the range of the weights threshold ($W_{r}$) were brought into a candidate condition. At this time, the center position was obtained in the UV space, and the pixels within the length of $W_{r}$ were searched around with the center position, the pixels within the range that met the conditions of Equations (3) and (4) were found, and $W_{r}$ was continuously increased and extended outside to obtain pixels suitable for the color temperature of the image. This benefits the proposed method in terms of better filtering out appropriate feature pixels for images with different color temperatures.

(1) $$D_{u}\left(x\right)=\underset{x}{\sum }\frac{\left|U\left(x\right)-M_{u}\left(x\right)\right|}{N}$$

(2) $$D_{v}\left(x\right)=\underset{x}{\sum }\frac{\left|V\left(x\right)-M_{v}\left(x\right)\right|}{N}$$

(3) $$\left|U\left(x\right)-\left(M_{u}\left(x\right)+D_{u}\left(x\right)\times \mathrm{sign}\left(M_{u}\left(x\right)\right)\right)\right|<W_{r}\times D_{u}\left(x\right)$$

(4) $$\left|V\left(x\right)-\left(M_{v}\left(x\right)+D_{v}\left(x\right)\times \mathrm{sign}\left(M_{v}\left(x\right)\right)\right)\right|<W_{r}\times D_{v}\left(x\right)$$

#### 2.2.2. Dynamical Feature Pixels Searching

**Figure 3:**
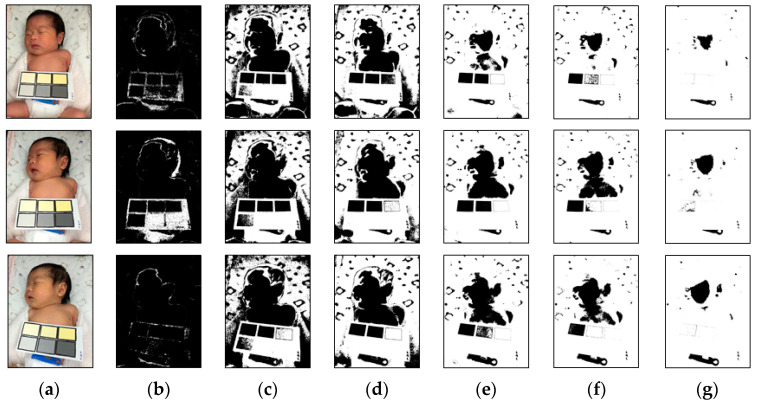
Results of the first processing step for the original images. (**a**) Original images with various color temperatures (32, 48, and 65 K from top to bottom). (**b**–**g**) The produced results with $W_{r}$ values of 0.1, 0.5, 0.7, 1.0, 1.2, and 1.5. The white regions in the images are the white candidates.

**Figure 4:**
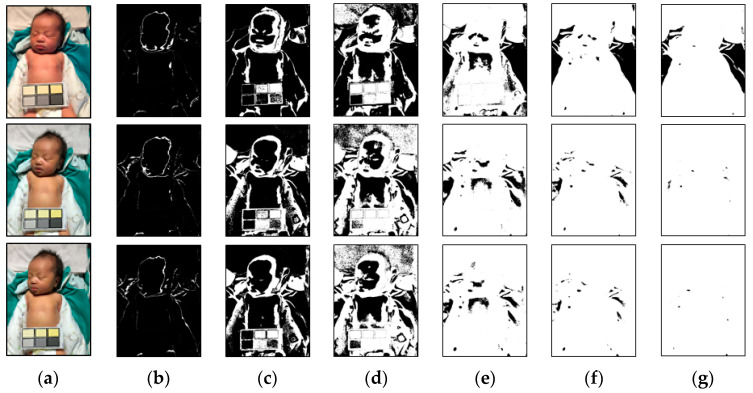
Results of the first step of processing of the original images. (**a**) Original images with various color temperatures (28, 48, and 65 K from top to bottom). (**b**–**g**) Results with $W_{r}$ values of 0.1, 0.5, 0.7, 1.0, 1.2, and 1.5. The white regions in the images are the white candidates.

The range of the said weights threshold ($W_{r}$) can be adjusted according to the parameter values. In this study, the test images had six different color temperatures, and each color temperature had a different image center and feature factor. In order to determine factors suitable for the image color temperature, the weights threshold ($W_{r}$) was adjusted by iteration. Under different environments and neonatal jaundice risks, the feature factors selected by different weights thresholds ($W_{r}$) varied, as shown in Figure 3 and Figure 4. After multiple tests and adjustments, it was found that if the initial parameter of the weights threshold ($W_{r}$) was 1 and the range was 0.5 to 1.5, the appropriate feature factor could be screened out in the case of six color temperatures for subsequent screening processes.

#### 2.2.3. Feature Pixels Optimization

**Figure 5:**
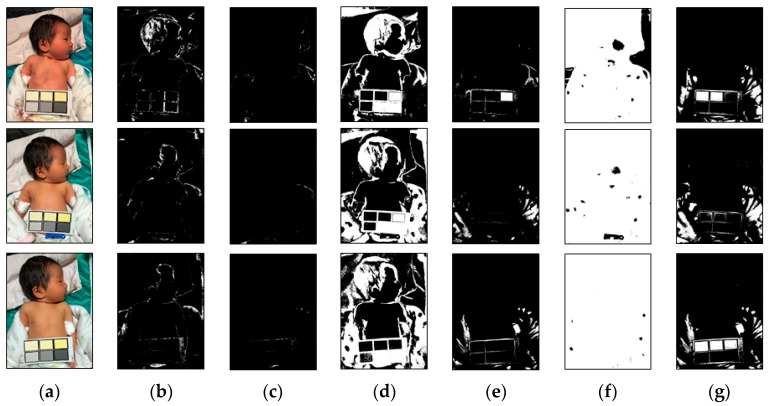
Results of original images after filtering. (**a**) Original images with various color temperatures (28, 48, and 65 K from top to bottom). (**b**,**d**,**f**) $W_{r}$ is 0.1, 0.5, and 1.5. $W_{p}$ is 0.1 for all color temperatures. (**c**,**e**,**g**) Remaining white points after filtering.

Figure 5 shows the selected feature factors of the percentage threshold ($W_{p}$) according to the preliminary results of different weights thresholds ($W_{r}$). The quantity of reserved feature factors meeting the weights threshold ($W_{r}$) condition varied with the images of six different color temperatures. In order to obtain the right quantity required for different color temperatures, the percentage threshold ($W_{p}$) was iterated, as the appropriate selection could be performed in different conditions. After the image had been tested numerous times, if the initial value was set as 0.1 and the range was 0.2 to 0.001, the correct feature factor could be selected from the reserved range of the weights threshold ($W_{r}$) in the case of six color temperatures (the actual white region color in the original image).

## 3. Experimental Results and Discussion

### 3.1. Quantitative Measurement of White Balance Method

**Figure 6:**
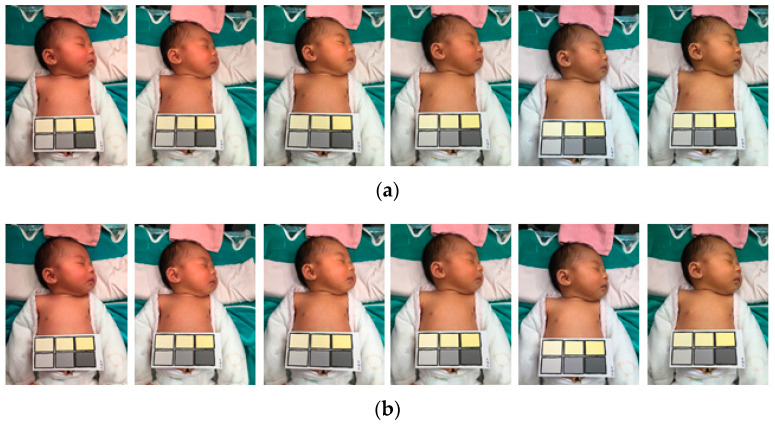
High risk of bilirubin level in control group. (**a**) Original images; (**b**) adjusted images with the proposed method.

**Figure 7:**
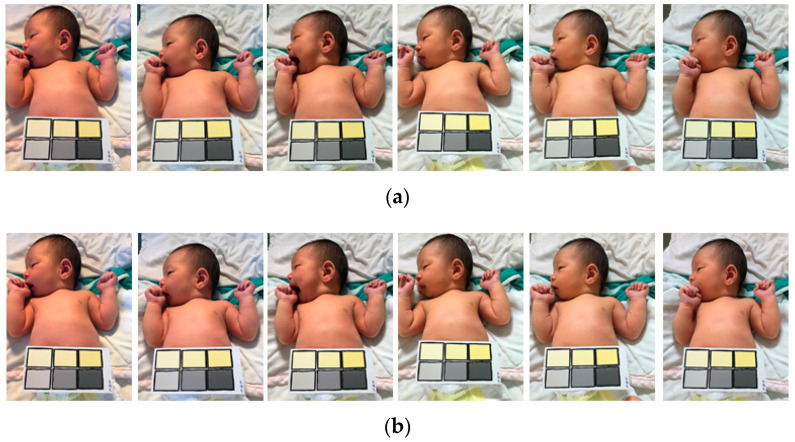
Low risk of bilirubin level in control group. (**a**) Original images; (**b**) adjusted images with the proposed method.

The collected samples were divided into two groups (i.e., high and low risk) according to the image color temperature (28 to 65 K). The proposed white balance method was used to adjust the sample images, which were then compared with the control group (i.e., the original images without automatic white balance adjustment). Different feature factors were extracted from each color temperature by the said two threshold parameters to restore the image color temperature. The proposed method could adjust different color temperature images to almost coincide with the color temperature image. Accordingly, the effects of ambient light sources and color temperature on diagnosis results can be reduced. As shown in Figure 6 and Figure 7, the proposed algorithm adjusted all images to the same color temperature, regardless of the original color temperature.

**Figure 8:**
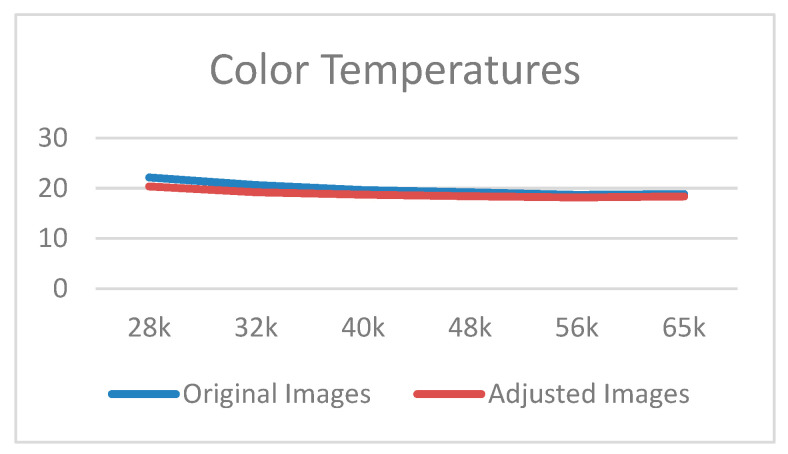
Curves at different color temperatures.

As presented in Table 2, the colors of the images were compared using the CIE2000 equation to determine the mean color difference. The performance of the proposed white balance method was verified by examining the reduction in color difference reduced under different color temperatures (28–65 K). Color difference reduction is crucial for medical image diagnosis because interfering factors such as different light sources should be avoided. As shown in Figure 8, when the adjusted results at different color temperatures are more similar, the flatter the curve, the more similar the adjusted color temperature values at different color temperatures; this means that the proposed system is powerful. White-balanced images are suitable for skin diagnosis because they display colors similar to those in the real world, thus enabling viewers to accurately identify and segment suitable images. The CIE2000 color difference test was used to compare the color differences between the original and white-balanced images. The results revealed that the color differences achieved using the proposed white balance method were lower than those of the original images. Because lower color differences indicate higher color temperature consistency, the results confirmed that the proposed algorithm could effectively improve the color consistency of the images (Figure 6 and Figure 7).

This study evaluated the chromatic components of the image with different color temperatures via CIEDE2000 [26]. This evaluation method is a new chromatic formula derived from the modified definition of CIE94 and the addition of five revised parameters:

(5) $$\Delta E_{00}^{*}=\sqrt{{\left(\frac{\Delta L^{\prime }}{K_{L}S_{L}}\right)}^{2}+{\left(\frac{\Delta C^{\prime }}{K_{C}S_{C}}\right)}^{2}+{\left(\frac{\Delta H^{\prime }}{K_{H}S_{H}}\right)}^{2}+R_{T}\frac{\Delta C^{\prime }}{K_{C}S_{C}}\frac{\Delta H^{\prime }}{K_{H}S_{H}}}$$

The CIEDE2000 value is between 0 and 100, and the smaller the value, the better the color fidelity.

### 3.2. Comparisons with the State-of-the-Art Approaches

**Figure 9:**
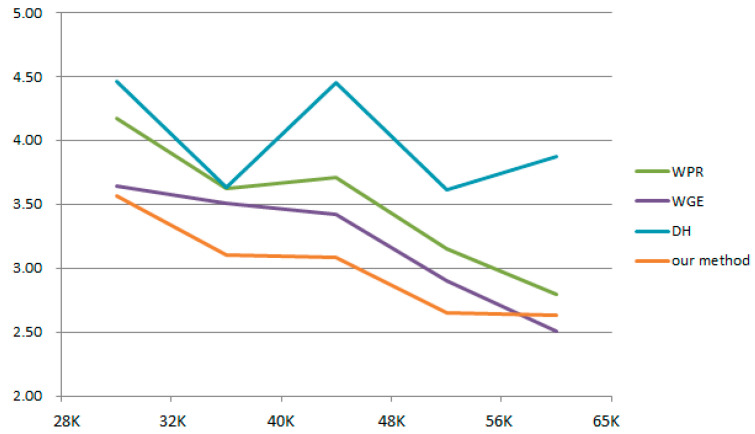
Curve diagram of different color temperature intervals.

Traditional white balance methods have prior hypotheses of their algorithms, and feature factors are screened based on different hypotheses. The proposed method was qualitatively compared with WPR [27], WGE [28], and DH [25]. It was found that in the case of different color temperatures, the image results of different methods were inconsistent. Table 3 shows that the resulting image adjusted at different color temperatures by our method has the minimum CIEDE2000 value, meaning the proposed method had similar adjustment results for different color temperatures. The proposed method could screen out the feature factor for each color temperature in the case of different color temperatures, thus reducing the number of unimportant feature factors. Figure 9 presents a visualization of the data. In comparison to the other methods, the proposed method had the minimum value among different color temperatures, and it had smoother fluctuations between different intervals than the other methods.

**Figure 10:**
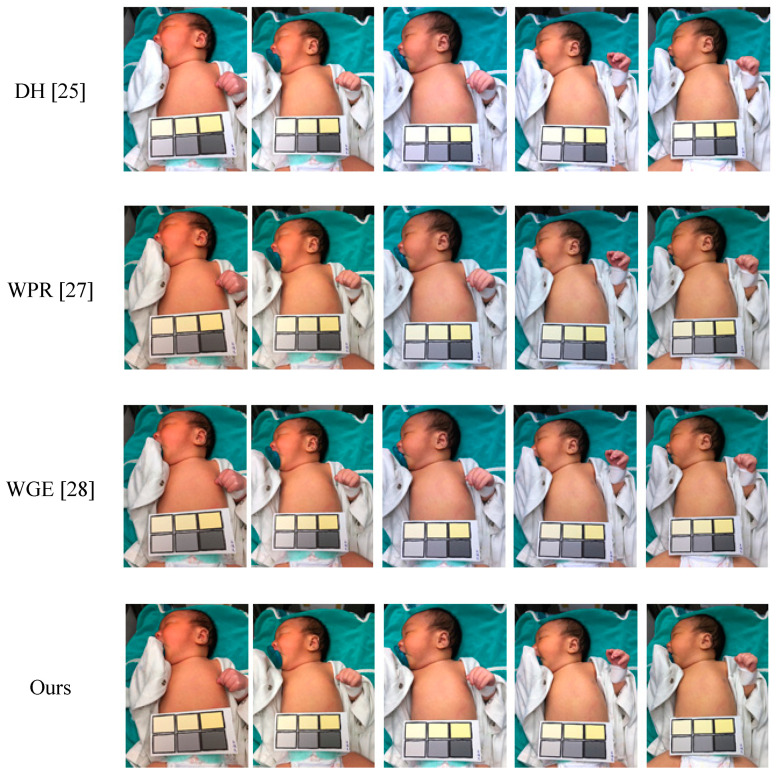
Adjusted resulting images of different color temperatures (32 K, 40 K, 48 K, 56 K, 65 K).

In the resulting images of different color temperatures, as shown in Figure 10, the severe color deviation caused by very low color temperatures (32 K) could not be restored correctly; however, the proposed method had better performance than the other methods for the other color temperatures. It was observed that the other methods had some color deviation in the 48 K and 56 K images, which may have resulted from the original image being captured at different color temperatures.

### 3.3. Research Contributions and Limitations

Neonatal jaundice detection systems usually contain numerous preprocessing programs, such as image color balance adjustment, skin area image capture, and image jaundice value detection, which bring different levels of difficulties to the application. Our study provided two main contributions. First, images could be adjusted at different color temperatures without a color chart by using the proposed method. Second, the feature factors for different color temperatures were selected by iteration, making the method favorable for feature screening in different color temperature images.

Our method is applicable to images of different color temperatures; however, if the original color of the image is changed by severe color deviation, the restoration will fail. For example, if an image is captured with a light source at a very low color temperature, the background and object of the image will be inclined to orange; on the contrary, a light source at a very high color temperature will result in a blue image. The background and object of the original image will fail to be restored to the actual colors under these effects.

## 4. Conclusions and Future Work

In research on neonatal jaundice detection systems, many researchers have tried to solve the problems in the image white balance process and have used color charts or feature factor screening mechanisms to accurately restore images. However, color charts have many uncertainties (such as color detection and whether the color chart slope is reflective or not). These effects may result in problems in determining the white balance of the color chart. The feature factor screening mechanism is similar to the experimentally compared methods; it is inapplicable to all color temperatures and must meet specific scene assumptions.

The data of the 38 participants collected in this experiment could be used as a reference in future studies. In future work, we will provide a sounder system flow, in which usable skin regions are extracted from the input image through color balance adjustments for jaundice value detection. The proposed white balance method could further improve the original system flow, thus allowing the neonatal jaundice value to be obtained simply and accurately as a reference for current neonatal jaundice detection systems.

## Figures and Tables

**Table 1:** The distribution of bilirubin levels and the number of group samples for high risk and low risk groups.

Group	Bilirubin Level (mg/dL)			Number
	AVG	MAX	MIN	
High risk	18.34	22.7	15.4	20
Low risk	8.6	14.3	0.6	18

**Table 2:** Color differences of the original and white-balanced images determined using the CIE2000 test under different color temperatures.

	Color Temperature					
	28 K	32 K	40 K	48 K	56 K	65 K
Origin Image	22.16	20.63	19.62	19.21	18.71	18.85
Our Method	20.36	19.18	18.70	18.39	18.17	18.35

**Table 3:** The results of CIEDE2000 among WPR [27], WGE [28], DH [25], and our methods.

Method	Color Temperature															
	28 K vs. 32 K	28 K vs. 40 K	28 K vs. 48 K	28 K vs. 56 K	28 K vs. 65 K	32 K vs. 40 K	32 K vs. 48 K	32 K vs. 56 K	32 K vs. 65 K	40 K vs. 48 K	40 K vs. 56 K	40 K vs. 65 K	48 K vs. 56 K	48 K vs. 65 K	56 K vs. 65K	Mean
WPR [27]	4.18	5.78	7.36	8.18	8.22	3.63	5.01	5.56	6.28	3.71	4.17	4.97	3.16	3.89	2.80	5.13
WGE [28]	3.65	5.15	6.21	6.65	6.62	3.51	4.46	4.71	4.70	3.43	3.40	3.78	2.91	2.93	2.51	4.31
DH [25]	4.46	5.61	6.41	6.66	7.36	3.64	4.34	4.61	5.57	4.45	4.65	5.44	3.62	3.83	3.88	4.97
Ours	3.57	4.50	5.56	5.97	6.04	3.11	3.95	4.23	4.44	3.09	3.10	3.44	2.66	2.65	2.64	3.93

## Data Availability

Data sharing is not applicable to this article.
